# Management of Residual Risk in Chronic Coronary Syndromes. Clinical Pathways for a Quality-Based Secondary Prevention

**DOI:** 10.3390/jcm12185989

**Published:** 2023-09-15

**Authors:** Simona Giubilato, Fabiana Lucà, Maurizio Giuseppe Abrignani, Laura Gatto, Carmelo Massimiliano Rao, Nadia Ingianni, Francesco Amico, Roberta Rossini, Giorgio Caretta, Stefano Cornara, Irene Di Matteo, Concetta Di Nora, Silvia Favilli, Anna Pilleri, Andrea Pozzi, Pier Luigi Temporelli, Marco Zuin, Antonio Francesco Amico, Carmine Riccio, Massimo Grimaldi, Furio Colivicchi, Fabrizio Oliva, Michele Massimo Gulizia

**Affiliations:** 1 Cardiology Department, Cannizzaro Hospital, 95126 Catania, Italy; famico64@gmail.com; 2 Cardiology Department, Grande Ospedale Metropolitano, AO Bianchi Melacrino Morelli, 89129 Reggio Calabria, Italy; fabiana.luca92@gmail.com (F.L.); massimo.rao@libero.it (C.M.R.); 3 Operative Unit of Cardiology, P. Borsellino Hospital, 91025 Marsala, Italy; 4 Cardiology Department, San Giovanni Addolorata Hospital, 00184 Rome, Italy; 5 ASP Trapani Cardiologist Marsala Castelvetrano Districts, 91022 Castelvetrano, Italy; nadiaing@hotmail.it; 6 Cardiology Unit, Ospedale Santa Croce e Carle, 12100 Cuneo, Italy; roberta.rossini2@gmail.com; 7 Sant’Andrea Hospital, ASL 5 Regione Liguria, 19124 La Spezia, Italy; giorgio.caretta@gmail.com; 8 Arrhytmia Unit, Division of Cardiology, Ospedale San Paolo, Azienda Sanitaria Locale 2, 17100 Savona, Italy; stefano.cornara@gmail.com; 9 De Gasperis Cardio Center, Niguarda Hospital, 20162 Milan, Italy; dimatteoirene@hotmail.it (I.D.M.); fabri.oliva@gmail.com (F.O.); 10 Department of Cardiothoracic Science, Azienda Sanitaria Universitaria Integrata di Udine, 33100 Udine, Italy; concetta.dinora@gmail.com; 11 Department of Pediatric Cardiology, Meyer Hospital, 50139 Florence, Italy; silvia.favilli@meyer.it; 12 Cardiology Unit, Brotzu Hospital, 09121 Cagliari, Italy; annapilleri@yahoo.it; 13 Cardiology Department, Papa Giovanni XXIII Hospital, 24127 Bergamo, Italy; andreawellsvabg@gmail.com; 14 Division of Cardiac Rehabilitation, Istituti Clinici Scientifici Maugeri, IRCCS, 28013 Gattico-Veruno, Italy; pierluigi.temporelli@icsmaugeri.it; 15 Department of Translational Medicine, University of Ferrara, 44121 Ferrara, Italy; zuinml@yahoo.it; 16 Department of Cardiology, West Vicenza Hospital, 136071 Arzignano, Italy; 17 CCU-Cardiology Unit, Ospedale San Giuseppe da Copertino Hospital, Copertino, 73043 Lecce, Italy; 18 Cardiovascular Department, Sant’Anna e San Sebastiano Hospital, 81100 Caserta, Italy; carminericcio8@gmail.com; 19 Department of Cardiology, General Regional Hospital “F. Miulli”, 70021 Bari, Italy; m.grimaldi@miulli.it; 20 Clinical and Rehabilitation Cardiology Unit, San Filippo Neri Hospital, 00135 Rome, Italy; furio.colivicchi@gmail.com; 21 Cardiology Department, Garibaldi Nesima Hospital, 95122 Catania, Italy; michele.gulizia60@gmail.com; 22 Heart Care Foundation, 50121 Florence, Italy

**Keywords:** chronic coronary syndromes, residual cardiovascular risk, secondary prevention, optimal medical therapy, multidisciplinary management, angina, percutaneous coronary intervention

## Abstract

Chronic coronary syndrome (CCS), which encompasses a broad spectrum of clinical presentations of coronary artery disease (CAD), is the leading cause of morbidity and mortality worldwide. Recent guidelines for the management of CCS emphasize the dynamic nature of the CAD process, replacing the term “stable” with “chronic”, as this disease is never truly “stable”. Despite significant advances in the treatment of CAD, patients with CCS remain at an elevated risk of major cardiovascular events (MACE) due to the so-called residual cardiovascular risk. Several pathogenetic pathways (thrombotic, inflammatory, metabolic, and procedural) may distinctly contribute to the residual risk in individual patients and represent a potential target for newer preventive treatments. Identifying the level and type of residual cardiovascular risk is essential for selecting the most appropriate diagnostic tests and follow-up procedures. In addition, new management strategies and healthcare models could further support available treatments and lead to important prognostic benefits. This review aims to provide an overview of the diagnostic and therapeutic challenges in the management of patients with CCS and to promote more effective multidisciplinary care.

## 1. Introduction

Coronary artery disease (CAD) remains a leading cause of mortality and morbidity worldwide. Recently, European [1] and American [2] guidelines have emphasized the importance of the clinical features of individuals at a higher risk of developing CAD, and the role of novel diagnostic tools in establishing a certain diagnosis. Importantly, the expression “stable” CAD has been removed and replaced with “chronic coronary syndromes (CCS)”. [3]. This change in nomenclature highlights the dynamic nature of CAD, which is characterized by a “silent” progression until the onset of clinical presentations, underlining that this clinical condition is only assumed to be stable. In fact, a chronic phase may be interrupted at any time by acute events, which can further clinically destabilize the course of the disease. International consensus statements suggest a stepwise approach to the diagnostic pathway, including the assessment of a patient’s history, symptoms, assessment of signs, risk factors, and comorbidities. Guidelines underscore the importance of a healthy lifestyle to prevent the onset of CAD and improve outcomes in patients with CCS. Optimal medical therapy (OMT), including antithrombotic and lipid-lowering therapy (LLT), is the first objective of CCS management for its proven prognostic efficacy. Only after intensification of pharmacological therapy should the assessment of myocardial ischemia, through both non-invasive and invasive tests, be considered to identify those patients who are likely to derive symptomatic benefit from myocardial revascularization. However, despite therapeutic advances in recent years, patients with CCS remain a population at a high risk for recurrent events [4]. Several mechanisms, including thrombotic, inflammatory, metabolic, and procedural factors, contribute to this residual risk and represent new targets for more effective and tailored secondary prevention strategies. 

The aim of this review is to address the diagnostic pathways of CCS and to provide an overview of the treatment challenges in managing residual cardiovascular risk in CCS patients to promote more effective secondary prevention strategies.

## 2. Chronic Coronary Syndromes: Nosographic and Epidemiological Aspects

Chronic coronary syndromes (CCS) encompass a wide spectrum of different clinical entities, mainly resulting from atherosclerotic plaque buildup in the wall of the coronary arteries (obstructive CAD) but also stemming from other pathogenetic mechanisms, such as epicardial vasospasm or microvascular coronary disfunction (non-obstructive CAD). From a nosographic perspective, the ESC guidelines have further classified CCS into six separate entities (Table 1) [1], facilitating the identification and categorization of these patients. 

From an epidemiological perspective, CCS represents an important and challenging public health issue. It has been reported that in the US, the incidence of CCS is approximately twice that of myocardial infarction (MI) and is expected to affect roughly 18% of adults by 2030 [5]. In contrast, the majority of the epidemiological and clinical evidence regarding the European population with CCS comes from the ongoing ESC EURObservational Research Programme (EORP) Chronic Ischemic Cardiovascular Disease Long Term (CICD LT) registry. This registry provides up-to-date information from 20 ESC countries regarding the management and outcomes of European patients with CCS [6]. The prevalence and mortality rate of patients with CCS varies slightly between European countries reflecting the existing differences in the treatment of acute coronary syndromes (ACS) and the promotion of secondary cardiovascular (CV) preventive strategies [6]. These differences are observed in the management of both women and elderly patients, as also previously reported in larger registries of patients with stable CAD. These groups are less likely to receive guideline-directed optimal medical therapy (OMT), resulting in poorer prognosis [7,8,9]. Conversely, the hospitalization rate of European patients with CCS remains high, with one in five patients being hospitalized for CV reasons < 1 year after CCS diagnosis [7]. Furthermore, the risk of CV events was higher among CCS patients with multiple non-cardiac comorbidities, including chronic kidney disease (CKD), chronic obstructive pulmonary disease (COPD), obesity, etc. Nevertheless, the rate of major adverse CV events (MACE) has substantially decreased in European patients with CCS, reflecting developments in their management [10]. Considering the recent introduction of a new CCS definition in clinical practice, in addition to the recent concomitant COVID-19 pandemic, which significantly limits the recruitment of patients in multicenter registries and trials, further analyses are necessary to better define the contemporary epidemiological features of CCS in different European member states, to improve the management and treatment of such patients. 

## 3. Residual Risk in Chronic Coronary Syndromes

Both the increased application of evidence-based therapy and the newer generation of drug-eluting stents (DES) have recently led to improvements in the treatment of patients with CAD. The revolutionary advancements have been linked with a significant reduction in the rates of recurrent MACE and stent thrombosis. However, despite the undoubtedly better approach, the risk of subsequent events remains very high, especially in patients with poly-vascular disease and comorbidities [11,12]. The high rate of recurrent CV events, despite a strong secondary prevention strategy, leads to the notion of residual ischemic risk. Clinical trials in patients with CCS have demonstrated a persistent risk of MACE of 2–4% per year [13,14,15]. Even in the most recent studies that introduced the proprotein convertase subtilisin/kexin type 9 inhibitors (PCSK9i) in the clinical arena, the occurrence of subsequent events in the treated arms remained around 9%, despite the drastic LDL cholesterol (LDL-C) reductions [16,17]. Moreover, real-world data from registries and observational studies demonstrated even more unfavorable outcomes: The REACH (Reduction of Atherothrombosis for Continued Health) registry reported an approximate 5% risk of 1-year recurrent CV events in patients with CAD or with multiple risk factors associated with atherothrombosis [18]. A comprehensive national Swedish registry, including over 108,000 post-MI patients, found that nearly 20% experienced a recurrent MACE in the year following the index ACS event. This residual CV risk persists and increases over time: In the Swedish registry, one in five patients who persisted stable in the first year after MI had a new recurrent event in the following 3 years [19]. The GRACE (Global Registry of Acute Coronary Events) registry, which recruited 3721 post-ACS patients from the United Kingdom (UK) and Belgium for a five-year follow-up period, presented a 13% occurrence of CV mortality and a 9.3% incidence of recurrent MI [20]. 

CCS patients with non-obstructive CAD (Category 5 according to the ESC Classification) also exhibit an elevated risk of MACE and all-cause mortality and deserve special attention [21]. Notably, women not only have a higher incidence of non-obstructive CAD but also have a worse prognosis than men [22].

Specifically, we have gained a deeper understanding of the prognosis of patients with microvascular angina. Several studies have shown that the detection of abnormal Coronary Flow Reserve (CFR) in patients with CCS without significant obstructive CAD is strongly associated with an increased risk of MACE in the long term [23,24]. Furthermore, Murthy VL et al. have shown that among diabetic patients, those without obstructive CAD but with an abnormal CFR have a CV mortality rate similar to those with obstructive CAD during 1.4 years of follow-up [25].

A large and growing body of research has demonstrated that around 6–8% of all MI occur in the absence of coronary artery obstruction. Myocardial infarction with non-obstructive CAD (MINOCA) is a heterogenous group of diseases with various potential etiologies, including coronary artery spasm, coronary thromboembolism, plaque disruption, spontaneous coronary artery dissection, and supply–demand mismatch [26]. Patients with a history of MINOCA exhibit a comparable, or only slightly lower, rate of recurrent MACE during follow-up when compared to post-MI patients with obstructive CAD, despite their younger age and fewer comorbidities [27]. The diagnosis of MINOCA is a working diagnosis in which intracoronary imaging techniques performed during the acute phase help to better evaluate coronary arteries that appear normal on angiography. In particular, optical coherence tomography (OCT), providing high-resolution, detailed images of the coronary arteries, plays a crucial role in understanding underlying etiopathogenetic mechanisms of MINOCA, such as plaque erosion, dissection, or thromboembolism. OCT’s ability to elucidate the etiology of MINOCA in more than half of patients holds significant therapeutic and prognostic implications. This allows for the customization of secondary prevention management strategies aimed at enhancing the overall prognosis of this CCS patient category [27,28]. In the future, the advancement of artificial intelligence-assisted techniques for characterizing coronary atherosclerotic plaques has the potential to improve the diagnostic power of both invasive and non-invasive approaches in suspected MINOCA cases [29].

When CCS coexists with heart failure (HF), the risk of recurrent CV events becomes even more pronounced. Previous studies have indicated that the development of HF over time in patients with stable CAD has significant prognostic implications [30,31]. More recently, findings from the CORONOR Registry have supported the notion that reduced left ventricular ejection fraction (LVEF) and a history of HF are the main causes of CV death in a modern population of patients with stable CAD who are extensively managed with guideline-directed medical therapy (GDMT) [32]. The CORONOR Registry also reported a 5.7% risk of hospitalization for HF over a 5-year period in this patient cohort. The study further highlighted that hospitalization for HF is a robust predictor of mortality with a 28% risk after 1 year and a 43% risk after 2 years [33].

**Figure 1:**
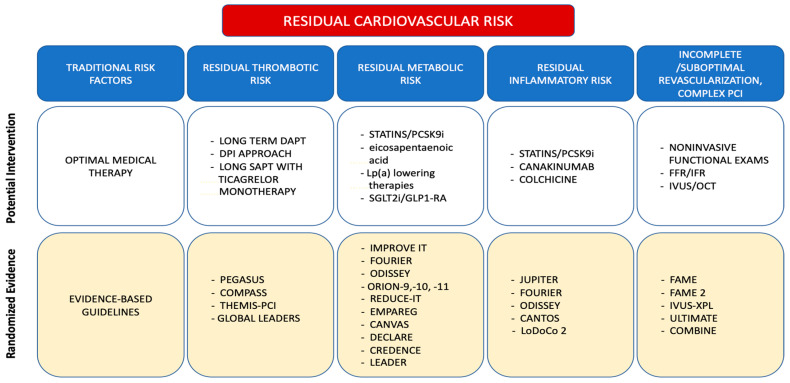
Pathways of residual CV risk, scientific evidence, and potential targeting interventions.

There are several elements contributing to the residual risk, including traditional CV risk factors, HF, CKD, and psychological and socio-cultural factors. However, other promoters of residual risk are beginning to emerge. These are related to thrombotic, metabolic, and inflammatory pathways that can contribute to the development of recurrent events and are often not adequately addressed in common clinical practice [34]. Furthermore, in revascularized CCS patients, other factors, such as incomplete/suboptimal revascularization or complex PCI, may contribute to a residual ischemic risk. Figure 1 shows pathways of residual CV risk in CCS patients.

### 3.1. Prognostic Stratification

The residual CV risk has been associated with some clinical characteristics (diabetes, prior ACS, stroke, HF, poly-vascular disease, extent of CAD, completeness of revascularization) and biomarkers, such as troponins, N-terminal pro-B-type natriuretic peptide (NT-proBNP), and C-Reactive protein (CRP) [35]. Identifying patients with high residual CV risk is crucial for the secondary prevention strategies.

Risk scores are useful bedside tools for a rapid prognostic definition. The Dual Antiplatelet Therapy (DAPT) score was developed from the DAPT Study to predict ischemic and bleeding risk in patients who underwent percutaneous coronary intervention (PCI). Patients with a DAPT score ≥2 have a high ischemic risk and a low bleeding risk and were found to benefit from prolonged DAPT beyond 12 months. On the other hand, patients with a DAPT score < 2 present a high bleeding risk and a low ischemic risk and were found to benefit from a shorter duration of DAPT [36]. Following the development of the DAPT score, many studies have tried to define its validity in different study populations with disappointing results. Chicharron et al. confirmed the ability of the DAPT score to identify an ischemic risk, detecting an approximately twofold higher incidence of CV events in patients with a DAPT score ≥2 [37]. In contrast, Ueda et al. showed that in an extensive Swedish registry, this score did not effectively discriminate between bleeding and ischemic risk [38]. In a recent analysis, including 100,211 post-PCI patients, a DAPT score ≥2 was able to accurately recognize patients at high ischemic and low bleeding risk [39].

More recently, the Predicting Bleeding Complication in Patients Undergoing Stent Implantation and Subsequent Dual Antiplatelet Therapy (PRECISE-DAPT) score, specifically designed for the prediction of bleeding, was introduced [40]. A recent meta-analysis involving over 50,000 patients established that a PRECISE DAPT score >25 was linked to an increased likelihood of major bleeding and an elevated risk of MACE [41].

Although the overlap between ischemia and bleeding is clear, Lindholm et al. demonstrated that as the number of CV risk factors increases, the risk of ischemic events increases more than the risk of bleeding. Furthermore, when patients with a history of bleeding are excluded, an increased number of CV risk factors is linked with a significant rise in ischemic events, with no significant difference in the rate of bleeding [42]. 

A 4-year analysis of the REACH registry demonstrated that a correct prognostic stratification cannot ignore the identification of some clinical predictors of future CV events. Subjects with atherothrombosis are extremely heterogeneous and consequently have a highly variable risk of events. This risk varies from 7% in patients with only CV risk factors, excluding diabetes, to 25% in individuals with a history of ischemic events and/or poly-vascular disease. Notably, patients with previous acute CV events have a higher residual risk compared to those with stable CAD, especially during the first year; in turn, individuals with stable CAD have a higher risk compared to those with CV risk factors but without atherosclerosis. Among all risk factors, diabetes has been confirmed to raise the ischemic risk, whereas, in the subjects with atherothrombosis, the presence of poly-vascular disease has been identified as the strongest independent risk factor for recurrent MACE [43]. 

Subsequent ischemic events may be due to the coronary artery that was originally treated or to other sites because of disease progression. In the previously mentioned Swedish registry, the risk of recurrent ACS due to an untreated or a non-culprit lesion was twice as high as the risk of recurrent ACS due to a previously stented lesion. Predictors of recurrent ACS from an untreated lesion were male sex, multivessel disease, and a longer interval between the index and the recurrent event [35].

Intracoronary imaging techniques, identifying lesions with features suggestive of plaque vulnerability, may be useful in the prognostic stratification of patients with CCS. The Providing Regional Observations to Study Predictors of Events in the Coronary Tree (PROSPECT) study enrolled 697 ACS patients who underwent three-vessel intravascular ultrasound (IVUS) imaging after PCI. During three years of follow-up, the recurrent events were equally attributable to culprit and non-culprit lesions. Common imaging-based characteristics of non-culprit lesions associated with recurrences were angiographically mild lesions with a large plaque burden, thin-cap fibroatheroma (<65 mm), and small luminal area [44]. In the Relationship between OCT Coronary Plaque Morphology and Clinical Outcome (CLIMA) Study, which involved 1003 patients who underwent OCT imaging of the untreated proximal left anterior descending (LAD) coronary artery, the simultaneous occurrence of four high-risk OCT plaque characteristics (lipid arc >180°, minimal lumen area < 3.5 mm^2^, minimum thickness of the fibrous cap < 75 µm, presence of macrophages) was linked to an increased MACE risk [45]. 

### 3.2. Therapeutic Targets

#### 3.2.1. Lifestyle

Improving healthy behaviors and medical adherence are the cornerstone of secondary CV prevention. In fact, a healthy diet, regular physical activity, stopping smoking, and maintaining an optimal body mass index (BMI) significantly reduce the recurrence of MACE, even after adjusting for guideline-recommended secondary prevention treatments [46,47,48]. Table 2 shows lifestyle recommendations.

Additionally, the ESC guidelines recommend cognitive-behavioral interventions to support CCS patients to adopt healthy behaviors, which may require a multidisciplinary team of experts (cardiologists, nurses, dieticians, general practitioners, physiotherapists, and psychologists), exercise-based cardiac rehabilitation, and the annual flu vaccination, particularly in patients older than 65 years and in those with HF [48,49,50,51].

#### 3.2.2. Psychosocial Risk Factors

The onset and progression of CAD are closely linked to psychosocial factors, including acute and chronic life stressors, low socioeconomic status, mental health disorders, depression, and inadequate sleep patterns. These factors are associated with the development and prognosis of CAD [52].

They exert their influence through a range of underlying mechanisms. Individuals struggling with compromised psychosocial well-being are more prone to adopting detrimental habits, such as smoking, alcohol/substance abuse, unhealthy diet, sedentary lifestyle, and inadequate adherence to prescribed medical regimens. This is all compounded by the fact that they often face limited access to health care resources. A large body of evidence indicates that stress triggers the activation of the inflammatory and neurohormonal systems, an elevation in blood pressure (BP), and a dysregulation of glucose metabolism. These effects can lead to clinical consequences, like myocardial ischemia, cardiac arrhythmias, atherosclerosis development, and the formation of more vulnerable coronary plaques [52,53]. Psychological factors have a significantly higher prevalence in certain demographic subgroups, including women, lower socioeconomic groups, and black individuals, contributing to disparities in CAD development and associated morbidity and mortality [54]. To improve the outcomes for CCS patients, it is imperative to systematically assess psychosocial risk factors and facilitate effective behavioral and pharmacological interventions by mental health professionals. These interventions are also critical in reducing social disparities in cardiovascular health [54,55,56].

#### 3.2.3. Blood Pressure Targets

Hypertension is the most common CV risk factor in CCS patients. Several studies have underscored the importance of anti-hypertensive treatment in CV secondary prevention, demonstrating that each 10 mmHg reduction in systolic BP is associated with an approximately 17% reduction in the rate of MACE [57,58]. Current ESC guidelines recommend a systolic BP target value of 120–130 mmHg in general CCS population and a target value of 130–140 mmHg in the elderly (Class I, LoE A) [59].

In symptomatic CCS patients, beta-blockers and calcium antagonists (CCBs) should be the drugs of choice, whereas in post-MI patients, beta-blockers and renin-angiotensin system (RAS) blockers should be used (Class I, LoE A). Treatment with both angiotensin-converting enzyme (ACE) inhibitors and angiotensin receptor blockers (ARBs) for hypertension is not recommended because it is linked with a higher incidence of major kidney adverse events (Class III, LoE A) [60].

#### 3.2.4. Residual Thrombotic Risk

For several decades, aspirin has been considered the basic component of antithrombotic therapy in patients with CCS. However, based on recent evidence, the latest ESC guidelines recommend a more potent antithrombotic approach in individuals exhibiting clinical, anatomic, and/or procedural features of high thrombotic risk without high bleeding risk (HBR) (Class II, LoE A) [1,61].

Two distinct and more intensive antithrombotic approaches have been demonstrated to significantly reduce recurrent MACE in this population: prolonged DAPT, which combines P2Y12 receptor antagonists, preferably ticagrelor 60 mg with aspirin [13], or a dual-pathway inhibition (DPI) approach, which combines a low-dose of rivaroxaban (2.5 mg) and aspirin [15]. Although the aforementioned guidelines do not indicate when one of the two strategies should be preferred over the other, a careful analysis of the trials testing these strategies allows us to identify the ideal candidates for the two pharmacological approaches. The best candidates for prolonged DAPT (>12 months) are non-HBR post-MI patients (within 2 years) with a moderate-high residual ischemic risk, who have not stopped DAPT for more than one year and who have tolerated DAPT for 12 months. Instead, CCS patients eligible for the DPI approach are non-HBR patients at any time after a MI with an additional ischemic risk factor, including those who have stopped DAPT for over one year, are high-risk patients without a prior MI, are patients with a prior stroke, or are patients with peripheral arterial disease (PAD) [62].

The Effect of Ticagrelor on Health Outcomes in Diabetes Mellitus Patients Intervention Study (THEMIS) evaluated DAPT with aspirin plus ticagrelor in stable diabetic patients without previous ischemic event. This study did not show a greater net clinical benefit of DAPT in these patients. Specifically, patients in the aspirin plus ticagrelor group exhibited a lower rate of MACE but a higher incidence of major bleeding compared to patients in the aspirin plus placebo group [63]. In THEMIS-PCI, which included only stable diabetic patients with previous PCI, the investigators demonstrated a lower incidence of MACE in the ticagrelor plus aspirin group. Major bleeding was significantly higher in the treatment arm, but fatal bleeding and intracranial hemorrhage were not significantly different between the two groups [64].

More recently, a new antithrombotic strategy involving long-term monotherapy with ticagrelor, after a short-term DAPT, has been tested in a population at high ischemic risk [65]. Although this so-called aspirin-free strategy is currently not yet recommended by the guidelines, it may become a valid pharmacological alternative in the future to achieve greater anti-ischemic efficacy without the trade-off of increased bleeding complications, particularly in HBR post-PCI patients who are not eligible for more intensive antithrombotic approaches. 

In patients with CCS and atrial fibrillation (AF) who underwent PCI, dual antithrombotic therapy with an oral anticoagulant (OAC), preferably Direct Oral Anticoagulants (DOACs), and clopidogrel is the default strategy recommended, with aspirin limited to the periprocedural phase, whereas only in the case of patients with additional clinical risk factors (diabetes, CKD, PAD, recurrent MI, etc.) and/or who have undergone complex PCI, triple antithrombotic therapy (TAT), which includes an OAC, clopidogrel, and ASA, should be continued for up to one month. Six months after elective PCI and lifelong in CCS patients, OAC alone is the recommended strategy [66]. For specific high-risk patients, dual antithrombotic therapy with OAC in combination with either clopidogrel or aspirin should be considered.

#### 3.2.5. Residual Metabolic Risk

- LDL:

In accordance with the ESC/EAS guidelines for the management of dyslipidaemias, the target of LDL-C levels should be lower than <1.4 mmol/L (<55 mg/dL) with a reduction of at least 50% from baseline (Class I, LoE A).

Moreover, an LDL-C target of <1.0 mmol/L (<40 mg/dL) may be considered for patients with CV disease who had a recurrent event within 2 years (Class IIb, LoE B) [67]. Management of dyslipidaemias includes lifestyle changes and LLT. Today, we have a broad therapeutic armamentarium to achieve these ambitious therapeutic goals, including high-intensity statins, ezetimibe, bempedoic acid, PSCK9i, and inclisiran. A high-intensity statin at the maximum tolerated dose represents the first-line therapy in CCS patients [68,69]. If statins fail to achieve LDL-C targets, a stepwise approach with ezetimibe first and then PSCK9i/inclisiran in combination with statins is recommended. For patients with intolerance to statins, the ESC guidelines [67] also recommend ezetimibe, bempedoic acid, and PSCK9i/inclisiran alone or in combination.

- Triglycerides (TG):

Hypertriglyceridemia is also linked with CV disease. Statin therapy is recommended in CCS patients with hypertriglyceridemia [triglycerides—TG—levels >2.3 mmol/L (>200 mg/dL)] (Class I, LoE B) [67]. Moreover, high-dose eicosapentaenoic acid (EPA) (2 g) taken b.i.d should be considered in high-risk individuals with persistent high TG levels (135–499 mg/dL) on statin treatment (Class IIa, LoE B). The Reduction of Cardiovascular Events with Icosapent Ethyl–Intervention (REDUCE-IT) trial, in fact, showed that in statin-treated patients with CAD or diabetes and mild–moderate hypertriglyceridemia (TG levels of 135 to 499 mg/dL) a high-dose EPA significantly reduced the incidence of ischemic events over a follow-up of 4.9 years. Indeed, the interventional group had a 25% relative risk reduction in the primary endpoint, a composite of CV death, MI, stroke, unstable angina, and coronary revascularization [70].

Despite this evidence, in contrast to LDL, current guidelines do not recommend a target for triglycerides [67].

- Lipoprotein(a) (Lp(a)):

Lp(a) is a genetically determined LDL variant that contains cholesterol, triglycerides, and an apolipoprotein(a) unit. Elevated Lp(a) levels are significantly linked to an increased risk of atherosclerotic CV diseases [71]. To date, there are no approved therapies specifically targeting Lp(a). Nevertheless, several agents aimed at lowering Lp(a), such as pelacarsen, olpasiran, and SLN360, are currently being evaluated in clinical outcome trials (Lp(a)-HORIZON; NCT04270760; NCT04606602). If proven effective, Lp(a) will soon become a new therapeutic target for reducing residual metabolic risk in CCS patients.

- Diabetes:

Individuals with CCS and diabetes represent a very high-risk population; therefore, ESC guidelines recommend a close monitoring of risk factors. For diabetic patients, BP should be 130/80 mmHg or below, whereas LDL-C < 1.4 mmol/L (<55 mg/dL) and reduced by at least 50% from the baseline. Additionally, a target glycated HbA1c level of less than 7% (<53 mmol/L) is recommended (Class I, LoE A) [72]. 

Until recently, none of the antidiabetic drugs could be shown to reduce MACE in diabetic patients, thus the results of randomized clinical trials (RCTs), which assessed the efficacy of two novel classes of glucose-lowering drugs, the sodium–glucose co-transporter-2 inhibitors (SGLT-2i) and the glucagon-like peptide-1 receptor agonists (GLP1-RA) marked a “new era” in diabetes treatment [73,74,75,76,77]. Notably, in these RCTs, these drugs provided additive benefits to GDMT (ACEi/ARB statins, statins, etc.). For these reasons, the ESC guidelines include strong recommendations for the use of SGLT2i and GLP-1 RA in patients with CCS and diabetes (Class I, LoE A) [72] and the new ADA guidelines recommend SGLT-2i and/or GLP1-RA for initial therapy, with or without metformin based on glycemic needs, in diabetic patients with high-risk features or established CV disease [78].

- Non-alcoholic fatty liver disease (NAFLD):

Nearly one-third of the adult population worldwide is affected by non-alcoholic fatty liver disease (NAFLD), which encompasses a broad spectrum of liver conditions. These range from simple steatosis to severe manifestations, such as non-alcoholic steatohepatitis (NASH). In some cases, NAFLD can progress into fibrosis and cirrhosis [79]. NAFLD, which is recognized as the hepatic component of the metabolic syndrome and closely associated with both obesity and diabetes, represents an emerging contemporary CV risk factor according to several studies [80,81,82]. There are a number of underlying mechanisms by which NAFLD may contribute to CAD development. These include insulin resistance, enhanced hepatic gluconeogenesis, atherogenic dyslipidemia, increased oxidative stress, and a prothrombotic state.

To date, specific treatments for NAFLD are lacking, and lifestyle interventions involving dietary modification, weight loss, increased physical activity and smoking/alcohol cessation are the primary recommended therapeutic strategies for patients with NAFLD [83]. A risk reduction in MACE as well as a modest improved NAFLD was shown in recent studies through the use of GLP1-Ras, improving glycemic control and supporting weight loss [84,85]. In addition, the ESSENTIAL study demonstrated the safety and efficacy of combination treatment with ezetimibe and rosuvastatin in reducing liver fat in patients with NAFLD [86]. Promising drug therapies targeting different stages of NAFLD are currently under investigation. However, many of these treatments have shown only moderate efficacy and, in some cases, their utility has been hampered by potential side effects and concerns about toxicity.

#### 3.2.6. Residual Inflammatory Risk

Systemic inflammation has emerged as an important player in the progression and destabilization of CV disease [87,88,89]. However, since the degree of inflammation as part of the residual ischemic risk varies among patients, it is important to have specific biomarkers and therapeutic targets to identify and treat patients at a high residual inflammatory risk to provide more personalized CCS care. As previously described, hs-CRP is the most extensively researched inflammatory marker linked to an elevated ischemic risk, independently of LDL levels. [87]. Statins can decrease hs-CRP (a pleiotropic effect), with the greater reduction in CV events observed in those who reached both the lowest LDL-C and hs-CRP levels (<2 mg/L) [90]. This effect has also been demonstrated when non-statin treatments, such as ezetimibe [91] or PCSK9i, were combined with statins [92].

The hypothesis that inflammation plays a key role in the pathogenesis of atherosclerosis was first demonstrated by the Canakinumab Anti-Inflammatory Thrombosis Outcome Study (CANTOS). In the CANTOS trial, 10,061 subjects with a history of MI, optimal LDL-C levels, and hs-CRP ≥ 2 mg/L were randomly assigned to receive OMT plus placebo or OMT plus canakinumab, an interleukin-1 beta blocker (IL-1β) without effect on LDL-C, BP, or platelets. Canakinumab (150 mg every 3 months) led to a significantly lower rate of CV events than placebo, but with a higher incidence of thrombocytopenia, neutropenia, and life-threatening infection [93]. However, the Food and Drug Administration (FDA), has not approved the use of canakinumab in patients with CAD. In contrast to CANTOS, the Cardiovascular Inflammation Reduction Trial (CIRT) demonstrated that low-dose methotrexate failed to reduce CV events in secondary prevention [94]. Recently, the Low-dose Colchicine 2 (LoDoCo2) trial showed that the anti-inflammatory drug colchicine in low doses (0.5 mg once daily) safely reduced CV events, including MI and the need for coronary revascularization in CCS patients on top of LDL-C lowering and antithrombotic treatments [95].

At present, despite the evidence supporting the role of inflammation in the development of atherosclerotic CV disease, effectively targeting inflammatory pathways in patients with CCS has proven challenging. Consequently, current guidelines do not recommend anti-inflammatory drugs for secondary CV prevention.

Novel anti-inflammatory interventions, including neutralization of IL-6 and the inflammasome, are still under investigation in several clinical trials.

## 4. Diagnostic Tests: What, When, and to Whom, with a View to Appropriateness and Rationalization of Resources

In CCS patients, the annual CV mortality rate describes the risk of an event. Thus, a CV mortality rate >3% per year identifies high-risk patients whereas a CV mortality rate < 1% per year identifies low-risk patients [96]. In addition, an annual risk assessment is warranted, even if the patient is asymptomatic. Several diagnostic tests may be useful for the diagnosis of CCS patients [1]. The main challenge in daily clinical practice is to ensure the right test for the right patient at the right time for an individualized diagnostic-therapeutic pathway in the context of healthcare resources rationalization. 

### 4.1. Pre-Test Probability (PTP)

The effectiveness of available diagnostic tests in diagnosing obstructive CAD depends on the prevalence of CAD in the population being studied. A simple predictive model, which includes only age, sex, and symptoms, can be routinely used to evaluate the pre-test probability (PTP) of obstructive CAD [97]. Based on more contemporary data [98,99], the new ESC guidelines on CCS have updated the method for estimating the PTP of obstructive CAD by significantly reducing the absolute values of the PTP [1]. The new PTP method increases the proportion of patients for whom diagnostic testing is not recommended. In fact, more patients fall into the PTP < 15% category, where the estimated CV mortality or MI rate per year is <1%, substantially reducing unnecessary diagnostic tests and costs. In addition, several PTP modifiers, such as CV risk factors, resting or exercise ECG abnormalities, LV dysfunction, and the presence of coronary calcium, have been introduced to better identify the clinical probability of CAD, particularly in patients with low PTP (5–15%) [1]. It should be kept in mind that each test has its own peculiarities to rule in or rule out CAD; therefore, clinicians should tailor the appropriate test according to patient’s PTP categories. Notably, stress ECG has limited diagnostic value across all levels of PTP; non-invasive stress imaging tests, in particular positron emission tomography (PET) and stress nuclear cardiac magnetic resonance (CMR), have better performance and may be preferred in patients with high PTP, whereas coronary computed tomography angiography (CTA) is the ideal technique to accurately exclude anatomic CAD in patients with a lower range of clinical likelihood of CAD [96,100].

### 4.2. Invasive Coronary Angiography (ICA)

The current gold standard test for the diagnosis of CAD is invasive coronary angiography (ICA). However, due to its invasive nature, ICA is associated with risks; therefore, it should be performed in patients who are likely to require coronary revascularization (patients with a high clinical probability or with severe symptoms despite OMT) [1]. Invasive functional test imaging, including fractional flow reserve (FFR) and instantaneous wave-free ratio (iFR), should be available to improve the diagnostic power of ICA and should be used to confirm or exclude uncertain diagnoses on non-invasive tests or to better assess stenosis severity before revascularization [101,102]. Therefore, current guidelines recommend non-invasive ischemia testing as a gatekeeper to ICA in individuals at low and intermediate risk for significant coronary stenosis [1]. 

### 4.3. Stress ECG

Stress ECG detects myocardial ischemia according to the electrocardiographic changes induced by exercise. However, its diagnostic power in detecting obstructive CAD is lower than that of other diagnostic imaging tests [96,103]. The prevalence of inconclusive stress ECG results is notably high, underscoring its limited efficacy in detecting myocardial ischemia. A stress ECG test should be avoided in patients with preexcitation, paced rhythm, and a left bundle branch block in whom ST-segment variations cannot be evaluated. Therefore, the dependence of the test on the patient’s physical fitness poses a significant limitation, potentially excluding individuals who are unable to undergo the procedure [104]. Stress ECG alone may be used as an alternative to detect ischemia if other imaging exams are unavailable, but caution is needed due to the risk of false-negative and false-positive results. Current guidelines recommend performing a non-invasive imaging or an anatomical test as the exam of choice for the diagnosis and for the follow-up of CCS patients after revascularization (IIa, LoE B) [1,2,103].

### 4.4. Non-Invasive Stress Imaging Tests

Stress echocardiography, in which the stressor may be represented by physical exercise or by pharmacological agents, is a useful non-invasive test for CAD diagnosis by detecting new or worsening ischemia-induced wall-motion abnormalities. Developments in contrast agents, image acquisition, and strain imaging have improved the diagnostic accuracy of this test. However, as stress echocardiography is an operator-dependent exam, its accuracy is highly dependent on the training and expertise of professionals performing the test to correctly interpret the results obtained.

The limitations of echocardiography in detecting exercise-induced kinetic abnormalities can be largely overcome, where available, using CMR. Conversely, both single-photon emission computed tomography (SPECT) and PET can identify myocardial ischemia by imaging regional myocardial tracer uptake, allowing the assessment of relative myocardial blood flow at rest and during stress. Overall, non-invasive functional tests exhibit a high sensibility and specificity for detecting flow-limiting obstructive CAD and are more accurate than ECG testing in defining the site of ischemia and in providing prognostic information [1,96,100]. The choice of non-invasive diagnostic exams is strictly dependent on patient characteristics (e.g., any contraindication to the administration of contrast medium), professional skills, and availability of such tests.

### 4.5. Coronary Computed Tomography Angiography (CCTA)

Coronary computed tomography angiography (CCTA) is a non-invasive imaging technique that uses an intravenous contrast agent to the visualize coronary artery wall with a high accuracy for the assessment of obstructive CAD. This diagnostic exam is not recommended (Class III, LoE C) for patients with high or irregular heart rates, extensive coronary calcification, severe obesity, or difficulty complying with breath-hold instructions that may interfere with the acquisition of high-quality images [1].

In cases of uncertain CCTA results or when CCTA is inconclusive, integrating functional data is advised. It is important to note that CCTA should not be used as a stand-alone follow-up test for patients with known CAD unless accompanied by functional information regarding myocardial ischemia. Recent technological developments have allowed the integration of three-dimensional CT-derived anatomical reconstructions with techniques able to predict FFR through computational fluid dynamics or machine learning. Although some studies have demonstrated a high diagnostic value of CCTA-based FFR [105], there are contrast data regarding its predictive value to detect CAD. A recent study of 2298 patients who underwent a CCTA-based FFR, reported a low positive predictive value (49%) [106]. Furthermore, Mittal et al. also highlighted the increased costs associated with CCTA-based FFR strategy compared with that of other stress imaging tests. The diagnostic landscape for CCS is characterized by the availability of multiple imaging modalities. However, the optimal selection of the most appropriate approach remains a challenge for most clinicians. Careful, case-by-case evaluation is essential to achieve optimal results and to rationalize the use of available resources. Several cost-effectiveness studies have been conducted to address this issue. In low-risk patients, anatomical analysis using CCTA has been shown to be cost-effective; functional strategy based on an echo stress test has shown comparable cost-effectiveness, whereas SPECT showed lower cost-effectiveness [107,108,109]. In contrast, in patients with an intermediate risk of CAD, functional tests, such as SPECT and stress echocardiography, seem to be the most cost-effective [109,110].

## 5. Pharmacological Management of Symptoms

Ideally, optimal treatment of CCS patients will not only improve prognosis but also symptoms and quality of life (QoL). Therefore, OMT must include effective antianginal drugs in combination with event prevention drugs. Current guidelines advocate for a tailored, stepwise approach in which antianginal therapy should be personalized, taking into account patient characteristics (such as BP, heart rate, LVEF, etc.), comorbidities, potential drug interactions, and specific underlying pathogenic mechanisms of angina [1]. 

Beta-adrenergic blockers (BBs) and/or calcium channel blockers (CCBs) represent the first-line treatment. The anti-ischemic effect of these classes of drugs is due to a reduction in heart rate, BP, and contractility, resulting in a reduced myocardial oxygen requirement. To optimize their efficacy, long-acting formulations should be preferred, and drugs should be carefully titrated. Beta-blockers and non-dihydropyridine CCBs are contraindicated in patients with sick sinus syndrome, severe bradycardia, advanced heart block, hypotension, and acute HF. If BBs and/or CCBs fail to successfully control angina symptoms, or if contraindications occur for these agents, several second-line drugs are available. These include long-acting nitrates and nicorandil, ivabradine, ranolazine, and trimetazidine. The addition of long-acting nitrates is a reasonable therapeutic option for patients who continue to experience symptoms despite taking BBs and CCBs, while short-acting nitrates can be used for the management of acute symptoms. Nitrates are contraindicated in patients taking phosphodiesterase inhibitors and in patients with hypertrophic obstructive cardiomyopathy (Class III, LoE B) [1]. Ivabradine and ranolazine are suitable therapeutic options for patients who develop low BP with usual anti-anginal drugs, as they do not exert vasoactive actions. Regardless of the type of initial treatment used, the patient’s response to therapy should be evaluated promptly.

## 6. Revascularization Strategy

In contrast to the clear data supporting the advantage of timely revascularization in reducing MACE and mortality in ACS patients [111,112], it is still controversial whether the revascularization strategy offers a prognostic benefit over OMT in the management of CCS patients.

The Clinical Outcomes Utilizing Revascularization and Aggressive Drug Evaluation (COURAGE) trial involved 2,287 patients with CCS and was the first trial to demonstrate that PCI plus OMT did not improve CV outcomes compared to OMT alone in patients with stable CAD. However, patients who underwent PCI were symptom-free and had an improved QoL after the intervention, though this difference was not maintained at 36 months [113].

Subsequently, in the 2nd Bypass Angioplasty Revascularization Investigation in Diabetes (BARI 2D) trial, which included 2368 diabetic patients with stable CAD, a revascularization strategy (PCI or Coronary Artery Bypass Graft-CABG) plus OMT was not superior to intensive OMT alone in reducing all-cause mortality at 5 years. However, in the CABG stratum, the incidence of MACE was significantly lower than in the OMT group, driven predominantly by a reduction in non-fatal MI [114]. 

The fractional flow reserve versus Angiography for Multivessel Evaluation 2 (FAME 2) trial showed that in CCS patients with angiographically documented coronary atherosclerosis, and at least one functionally significant stenosis (fractional flow reserve (FFR) ≤ 0.80), FFR-guided PCI with DES resulted in a significantly lower rate of death, non-fatal MI, or urgent revascularization compared to OMT alone. The benefit of FFR-guided PCI over OMT was driven by a significantly lower need for urgent revascularization in the PCI group and was sustained at 5 years of follow-up. This strategy has also shown a significant improvement in symptoms and QoL [115]. The FAME 2 trial established that the detection of significant ischemia, identified by a positive FFR test, allows a better risk stratification of patients with CCS before performing a PCI. Of note, in the subsequent FAME 3 trial, FFR-guided PCI with currently used DES failed to meet noninferiority compared with CABG among patients with three-vessel CAD.

The Objective Randomized Blinded Investigation with Optimal Medical Therapy of Angioplasty in Stable Angina (ORBITA) trial showed no significant improvement in exercise time at 6 weeks in patients with stable CAD undergoing PCI compared to the placebo procedure [116]. Although the scientific debate generated by this study underlines the importance of the indication for PCI in CCS patients, its impact on clinical practice and guidelines does not appear to be significant considering the important limitations of this study. These limitations include inadequate statistical power to demonstrate clinical endpoints, a small sample size, and a short follow-up period. 

Finally, the International Study of Comparative Health Effectiveness with Medical and Invasive Approaches (ISCHEMIA) trial was designed to overcome the limitations of previous trials. None of the previous trials were blinded; they enrolled patients with only mild ischemia whose coronary anatomy was known prior to randomization, raising the possibility of selection and referral bias. Moreover, patients in most of these trials were not treated with the current standard of care, which includes aggressive OMT, FFR-guided PCI, and the use of the latest generation of DES. The ISCHEMIA trial enrolled 5179 patients with moderate to severe ischemia at baseline prior to ICA. Coronary CTA was performed in all patients without renal dysfunction to exclude left main disease and non-obstructive CAD. Patients were randomized to receive OMT or invasive strategy plus OMT (revascularization by PCI or CABG, as clinically determined). This trial failed to demonstrate significant differences between the conservative and invasive strategy in the primary endpoint (a combination of CV death, MI, hospitalization for UA, hospitalization for HF, or resuscitated cardiac arrest). However, the invasive treatment strategy is associated with improved symptom control and QoL at the end of the trial [117].

More recently, an interim analysis of the ISCHEMIA EXTENDED, an observational study, including 4825 of the original ISCHEMIA trial participants, showed no difference in all-cause mortality between the two approaches. However, the invasive strategy demonstrated a lower CV mortality rate but a higher non-CV mortality rate during the 5.7 years of follow-up [118]. Table 3 shows the key characteristics and results of studies assessing OMT versus revascularization strategy in patients with CCS.

The decision to perform complete coronary revascularization in patients with ACS and evidence of multivessel CAD remains a matter of debate. Complete revascularization appears to be associated with a better outcome, with a reduction in both new revascularizations and hard clinical events [119].

Current ACC/SCAI/AHA guidelines for coronary artery revascularization suggest the use of FFR or iFR to assess angiographic, intermediate coronary lesions only in the presence of stable CAD (Class I, level of evidence A), but their role in ACS patients has not been clearly addressed [120]. The FLOWER-MI (FLOW Evaluation to Guide Revascularization in Multi-vessel ST-elevation Myocardial Infarction) trial compared angiography-guided and FFR-guided complete revascularization in STEMI patients with multivessel disease. The authors demonstrated that an FFR-guided strategy had no significant advantage over an angiography-guided strategy with respect to the primary endpoint (composite of death, MI, and urgent revascularization at 1 year) [121]. More recently, a comprehensive network meta-analysis of 11 RCTs comparing FFR and angiography in this setting concluded that complete revascularization of non-culprit stenosis was associated with a lower incidence of adverse events compared with culprit-only revascularization, but FFR guidance was not superior to angiography guidance in reducing MACE [122]. 

Some studies have investigated the best strategy in CCS patients with reduced LVEF. In the Surgical Treatment for Ischemic Heart Failure (STICH) trial, patients with extensive CAD and LV systolic dysfunction were randomly assigned to initial OMT or CABG. At the 10-year follow-up, CABG plus OMT demonstrated a prognostic benefit versus OMT alone [123]. The REVascularization for Ischemic VEntricular Dysfunction (REVIVED)-BCIS2 trial, the first powered trial to assess the efficacy and safety of PCI in patients with severe ischemic cardiomyopathy and evidence of myocardial viability, did not show a significant benefit of multivessel PCI versus OMT over 3.4 years of follow-up [124]. However, the failure of an invasive strategy in this setting may have been due to less extensive CAD, small sample size, and shorter follow-up.

Based on this evidence, the management of patients with CCS should always start with an aggressive contemporary OMT, and only after functional assessment of CAD should an individualized revascularization strategy be considered if the conservative strategy fails or is in subgroups of patients in whom a prognostic benefit has been demonstrated. Current guidelines suggest a tailored approach in which the decision to revascularize each individual patient by PCI or CABG should be established based on symptoms and QoL despite OMT, prognostic indicators (ischemic area >10% LV or ischemic cardiomyopathy with LVEF ≤ 35%), evidence of ischemia, and invasive assessment of CAD severity (FFR ≤ 0.80 or iwFR ≤ 0.89 in a major coronary vessel) [1].

While the prognostic significance of invasive methods for functionally assessing coronary lesions in ACS patients remains uncertain, recent years have seen growing evidence supporting the prognostic value of the use of intracoronary imaging techniques in complex PCI, regardless of clinical CAD presentation (ACS or CCS). In particular, various studies and meta-analyses have shown that IVUS-guided complex PCI, when compared to conventional angiography-guided PCI, leads to a significantly reduced rate of long-term MACE [125,126,127,128].

Regarding antiplatelet therapy in CCS patients undergoing elective PCI, the DAPT with clopidogrel plus aspirin remains the standard of care. In fact, trials testing the more potent P2Y12 inhibitors, such as prasugrel or ticagrelor, have not demonstrated their superiority over clopidogrel in this specific patient population [129,130].

## 7. Follow-Up Strategies and Care Pathways: From the Hospital to the Community Care

**Figure 2:**
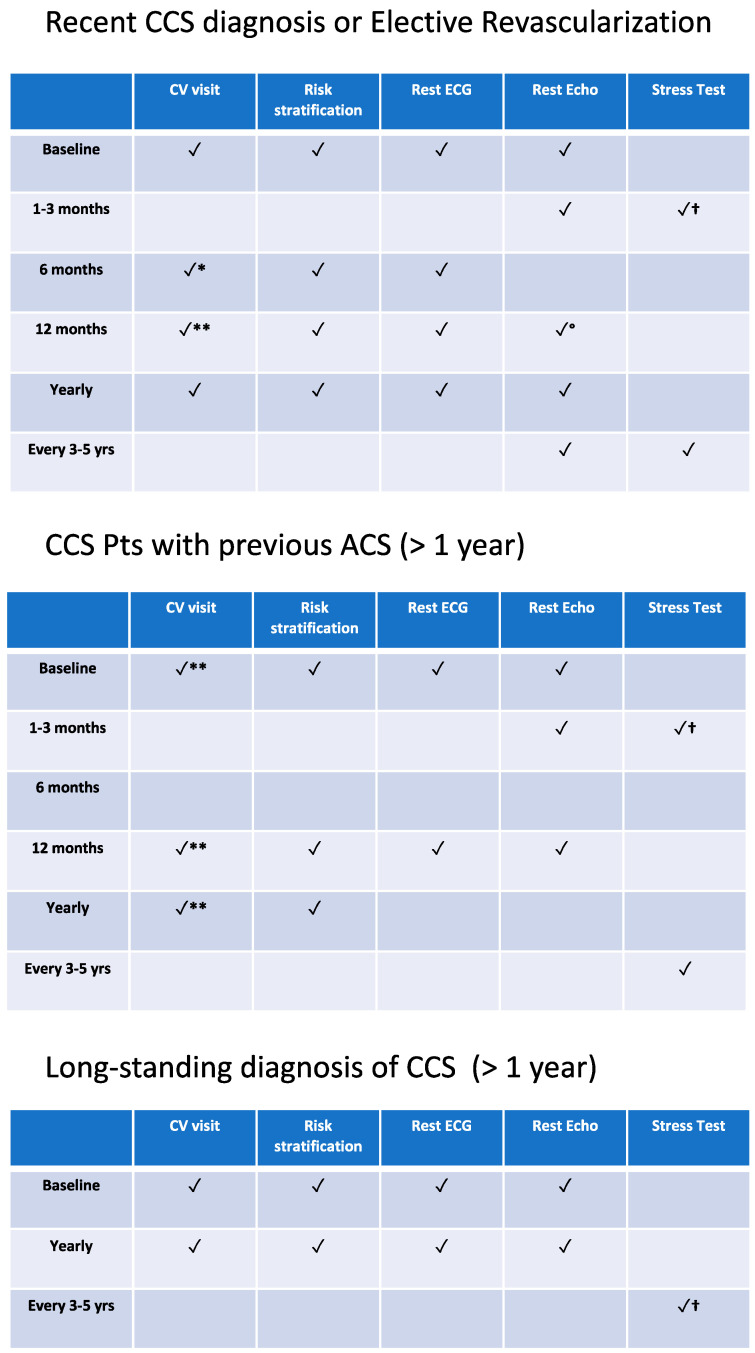
Model for the management of CCS patients. Modified from Knuuti J et al., EHJ 2020 [1]. * Time for decision-making on DAPT/SAPT in post-PCI patients; ** time for decision-making on long term antithrombotic therapy according clinical and procedural residual ischemic risk; † at any time to investigate changes in symptoms and/or functional status; invasive coronary angiography only for patients with symptoms despite OMT or with moderate/severe ischemia on non-invasive stress tests. ° In pts with previous abnormal rest echo. CV: cardiovascular; ECG: electrocardiogram; Echo: echocardiography.

In clinical practice, even asymptomatic CCS patients require a regular clinical follow-up by a CV professional to evaluate any change in the patient’s residual CV risk, adherence to lifestyle recommendations and pharmacological therapy, or the occurrence of comorbidities that may affect therapy and CV outcomes. Identifying the patient’s clinical risk is also important to ensure that the right patient receives the right instrumental follow-up modality. Resting echocardiography should be performed annually if previously abnormal or every 3/5 years if previously normal. In patients who have undergone revascularization, an early echocardiographic assessment may be useful 1-3 months after the procedure [1]. In general, the routine use of inductive ischemia tests is not advised, regardless of the presence or absence of symptoms, even in cases of previous revascularization. Non-invasive stress exams may be considered 1 year after PCI and 5 years after CABG or every 3/5 years to assess silent ischemia, preferably using non-invasive stress-imaging techniques (Class IIb, LoE C) [1]. Among high-risk post-PCI patients with incomplete or suboptimal revascularization, early evaluation (3 months after the procedure), preferably with an imaging stress test, could be useful. In addition, an early evaluation (1-3 months after the procedure) has been suggested, in particular, in clinical settings of CCS to establish a reference for subsequent follow-up. A model for the management of CCS patients, tailoring examinations and follow-up visits according to CCS categories and their level of risk is shown in Figure 2.

Coronary CTA should not be used as a routine follow-up test for CCS patients, whereas ICA, with FFR/iFR when necessary, is the test of choice for high-risk patients according to non-invasive test results and for patients with severe symptoms despite OMT.

It is critical that after hospitalization for ACS or elective PCI, the follow-up modality is established at the time of discharge based on the patient’s risk level.

To reduce the recurrence of CV events, the ESC guidelines recommend that patients with CAD be discharged according to a structured modality for the optimal management of the post-discharge pathway (Class IIa, LoE B) [49]. In addition, a recent Cochrane analysis demonstrated that discharge planning can reduce unplanned readmissions and improve the coordination of post-discharge services [131]. To this end, the discharge letter is a key component of the transition between hospital and community care.

## 8. Multidisciplinary and Multi-Professional Management Aspects

Modern secondary prevention of CV is evolving into a partnership between CCS patients and healthcare professionals to improve prognosis through appropriate medications, interventions, and lifelong healthy lifestyle behaviors. This collaborative approach requires an integrated, multidisciplinary team approach, including (in both hospital and community settings) cardiologists, nurses, dieticians, physiotherapists, psychologists, general physicians, diabetologists, nephrologists, geriatricians, and others, who can provide holistic and personalized care to patients. To achieve this, professionals should have sufficient knowledge, expertise, and tools to manage the complex, specialized cardiac needs of a patient who often has multiple comorbidities. Today, digital medicine through new telemedicine and telemonitoring technologies can help improve an integrated, patient-centered approach among the different professionals involved in managing CCS patients, optimizing the use of healthcare resources, and improving the prognosis of this patient population.

It is also essential that national core components of cardiac support and secondary prevention are structured to ensure that CCS patients have equal access to the best evidence-based care. This requires an integrated network between hospitals and the community on a regional basis to guarantee continuity of care and patient empowerment.

## 9. Conclusions

The management of patients with CCS remains a diagnostic and therapeutic challenge. Despite significant therapeutic advances in recent years, patients with CCS continue to experience a high rate of recurrent CV events [132]. As a result, the concept of stable CAD has been reevaluated and the notion of residual CV risk has been introduced. This risk persists despite the use of the best available evidence-based secondary prevention strategies [133]. To significantly reduce this risk, comprehensive strategies should be employed, including: (a) a selection of the most appropriate diagnostic tools; (b) a personalized assessment of residual risk, taking into account contemporary, non-traditional risk factors and their evolution over time; (c) tailoring of therapeutic approaches, both pharmacological and non-pharmacological, to individual risk profiles; (d) an establishment of optimal and individualized follow-up protocols according to the CCS categories and their risk level; (e) implementing a multidisciplinary patient-centered approach to care that can incorporate innovative telemedicine and telemonitoring technologies; (f) promoting integrated management between hospital and community care; (g) incorporating non-pharmacological interventions to improve CV health education.

## Figures and Tables

**Table 1:** CCS categories according to the ESC 2019 guidelines [1].

CCS Categories	Description
1	Patients with supposed CAD and “stable” symptoms
2	Patients with new onset of heart failure or left ventricular dysfunction and suspected CAD
3	Asymptomatic and symptomatic patients < 1 year after an ACS or revascularization
4	Patients >1 year after angina diagnosis or revascularization
5	Symptomatic patients with suspected vasospastic or microvascular disease
6	Asymptomatic patients in whom CAD is discovered at screening

ACS: acute coronary syndrome CAD: coronary artery disease; CCS: chronic coronary syndromes.

**Table 2:** Lifestyle ESC Guidelines recommendations for CCS patients [1,49].

	Intervention	Relative Risk Reduction %
**Stopping Smoking**	Use the ‘Very brief advice’ for smoking cessation: - ASK: establishing and recording smoking status - ADVISE: advising on the best methods of stopping - ACT: offering help	36 (mortality)
**Healthy diet**	High in vegetables, fruits, and grains. Saturated fats < 10% of total intake. Limit alcohol to <100 g/week or 15 g/day.	31 (MACE)
**Physical activity**	30–60 min of moderate-intensity aerobic activity ≥5 days per week.	27 (mortality)
**Weight loss**	BMI ≤ 25 kg/m^2^	33 (MACE)

**Table 3:** Principal contemporary studies assessing OMT versus the revascularization strategy in patients with CCS.

	COURAGE [113]	BARI 2D [114]	FAME 2 [115]	ISCHEMIA [117]
Publication year	2007	2009	2014	2020
N° pts	2287	2368	1220	5279
Follow-up (yrs)	4.6	5	2	3.2
Documentation of ischemia required?	No	No	No	Yes, >10%
CTA performed before enrollment?	No	No	No	Yes
Enrollment before ICA?	No	No	No	Yes
Contemporary conservative strategy?	No	No	Yes	Yes
Contemporary invasive strategy?	No, only PCI, no DES	No, only 35% DES, 10% no stent	Yes, DES, FFR	Yes, DES, FFR
Main Results	Neutral QoL improvement	Neutral Less CV events in CABG arm	Neutral Less need for urgent revascularization	Neutral QoL improvement

° in patients with previous abnormal rest echo; OMT: optimal medical therapy; CTA: computed tomography angiography; ICA: invasive coronary angiography; DES: drug-eluting stent; FFR: fractional flow reserve.

## Data Availability

Data sharing is not applicable to this article as no new data were created or analyzed in this study.
